# Supplementary material for: IsoBayes: a Bayesian approach for single-isoform proteomics inference
Source: bioRxiv. 2024 Jun 11:2024.06.10.598223. Preprint. [Version 1] doi: 10.1101/2024.06.10.598223 (PMC11195044; doi:10.1101/2024.06.10.598223)
Supplement: Supplement 1 [file media-1.pdf]

Supplementary material
IsoBayes: a Bayesian approach for single-isoform proteomics inference

Jordy Bollon[1,2], Michael R Shortreed[3], Ben T Jordan[4], Rachel Miller[3], Erin Jeffery[5], Andrea Cavalli[1,6], Lloyd M Smith[3], Colin Dewey[7], Gloria M Sheynkman[5*], and Simone Tiberi[8*]

[1]*Computational and Chemical Biology, Italian Institute of Technology, CMP^{3}VdA, Aosta, Italy.*
[2]*Astronomical Observatory of the Autonomous Region of the Aosta Valley (OAVdA), Nus, Italy.*
[3]*Department of Chemistry, University of Wisconsin-Madison, Madison, WI, USA.*
[4]*Frederick National Laboratory for Cancer Research, Frederick, MD, USA.*
[5]*Department of Molecular Physiology and Biological Physics, University of Virginia, Charlottesville, VA, USA.*
[6]*Centre Européen de Calcul Atomique et Moléculaire, École Polytechnique Fédérale de Lausanne, Lausanne, Switzerland.*
[7]*Department of Biostatistics and Medical Informatics, University of Wisconsin, Madison, WI, USA.*
[8]*Department of Statistical Sciences, University of Bologna, Bologna, Italy.*
[*] e-mail: gs9yr@virginia.edu and Simone.Tiberi@unibo.it

1 Supplementary Tables

1.1 Simulation study

Cell line	Protease	AUC	log10-corr	0.95 CI coverage	Abundance absent iso	Abundance present iso
jurkat	ArgC	0.91	0.86	0.98	0.69	5.73
jurkat	AspN	0.93	0.89	0.98	0.60	8.73
jurkat	Chym	0.87	0.83	0.98	0.71	4.41
jurkat	GluC	0.93	0.90	0.98	0.59	9.14
jurkat	LysC	0.95	0.92	0.99	0.48	11.84
jurkat	Trypsin	0.95	0.92	0.98	0.47	11.30
WTC-11	AspN	0.92	0.82	0.96	0.82	7.28
WTC-11	Chymo	0.89	0.78	0.94	1.16	9.62
WTC-11	LysC	0.93	0.86	0.97	0.65	7.43
WTC-11	Trypsin	0.93	0.88	0.98	0.70	7.26
Average		0.92	0.87	0.98	0.69	8.28

Supplementary Table 1: Summary results, from the simulation study, for *IsoBayes* fit without mRNA abundances. "Abundance present iso" and "Abundance absent iso" indicate the estimated average abundance for protein isoforms which were actually simulated to be present and absent, respectively.

Cell line	Protease	AUC	log10-corr	0.95 CI coverage	Abundance absent iso	Abundance present iso
jurkat	ArgC	0.97	0.95	0.99	0.24	6.01
jurkat	AspN	0.97	0.97	0.99	0.16	8.97
jurkat	Chym	0.96	0.96	0.99	0.16	4.91
jurkat	GluC	0.98	0.97	0.99	0.16	9.40
jurkat	LysC	0.98	0.98	0.99	0.15	11.98
jurkat	Trypsin	0.98	0.98	0.99	0.13	11.46
WTC-11	AspN	0.97	0.96	0.99	0.17	7.62
WTC-11	Chymo	0.97	0.97	1.00	0.18	10.13
WTC-11	LysC	0.97	0.97	0.99	0.17	7.68
WTC-11	Trypsin	0.96	0.96	0.99	0.18	7.46
Average		0.97	0.97	0.99	0.17	8.56

Supplementary Table 2: Summary results, from the simulation study, for *IsoBayes* fit with mRNA abundances. "Abundance present iso" and "Abundance absent iso" indicate the estimated average abundance for protein isoforms which were actually simulated to be present and absent, respectively.

1.2 Real data - All isoforms

Cell line	Protease	*IsoBayes_mRNA*	*IsoBayes*	*EPIFANY*	*PIA*	*Fido*
jurkat	ArgC	0.84	0.80	0.73	0.65	0.69
jurkat	AspN	0.86	0.82	0.75	0.67	0.7
jurkat	Chym	0.85	0.78	0.73	0.63	0.67
jurkat	GluC	0.85	0.81	0.76	0.67	0.69
jurkat	LysC	0.86	0.84	0.78	0.71	0.73
jurkat	Trypsin	0.86	0.84	0.77	0.69	0.71
WTC-11	AspN	0.89	0.81	0.70	0.62	0.61
WTC-11	Chym	0.85	0.75	0.76	0.60	0.60
WTC-11	LysC	0.88	0.84	0.84	0.67	0.75
WTC-11	Trypsin	0.87	0.84	0.85	0.68	0.68
Average		0.86	0.81	0.77	0.66	0.68

Supplementary Table 3: Area under the curve (AUC) for the detection of protein isoforms, for every method in each real dataset.

Method	*jurkat*	*WTC-11*
IsoBayes_mRNA	0.87	0.90
IsoBayes	0.88	0.90

Supplementary Table 4: Correlation between log10 estimated protein gene abundances (i.e., log10(abundance + 1)), and those found in the validation set. In each cell line, we considered results from all protesease.

1.3 Real data - Isoforms without unique peptides

Cell line	Protease	*IsoBayes_mRNA*	*IsoBayes*	*EPIFANY*	*PIA*	*Fido*
jurkat	ArgC	0.81	0.73	0.67	0.55	0.66
jurkat	AspN	0.82	0.75	0.69	0.57	0.67
jurkat	Chym	0.80	0.70	0.66	0.53	0.63
jurkat	GluC	0.80	0.73	0.68	0.57	0.65
jurkat	LysC	0.82	0.78	0.73	0.62	0.71
jurkat	Trypsin	0.81	0.76	0.71	0.60	0.69
WTC-11	AspN	0.83	0.69	0.64	0.53	0.60
WTC-11	Chym	0.82	0.66	0.66	0.51	0.58
WTC-11	LysC	0.85	0.75	0.72	0.57	0.69
WTC-11	Trypsin	0.83	0.77	0.72	0.60	0.69
Average		0.82	0.73	0.69	0.56	0.66

Supplementary Table 5: Area under the curve (AUC) for the detection of protein isoforms, for every method in each real dataset, computed on the subset of protein isoforms solely associated to shared peptides.

1.4 Real data - Isoforms from multi-isoform genes

Cell line	Protease	*IsoBayes_mRNA*	*IsoBayes*	*EPIFANY*	*PIA*	*Fido*
jurkat	ArgC	0.83	0.78	0.74	0.61	0.73
jurkat	AspN	0.84	0.8	0.75	0.64	0.73
jurkat	Chym	0.81	0.74	0.7	0.59	0.68
jurkat	GluC	0.82	0.78	0.74	0.64	0.72
jurkat	LysC	0.85	0.83	0.78	0.7	0.77
jurkat	Trypsin	0.84	0.82	0.77	0.69	0.76
WTC-11	AspN	0.86	0.75	0.7	0.59	0.64
WTC-11	Chym	0.83	0.7	0.68	0.55	0.61
WTC-11	LysC	0.87	0.81	0.79	0.65	0.74
WTC-11	Trypsin	0.86	0.83	0.81	0.67	0.71
Average		0.84	0.78	0.75	0.63	0.71

Supplementary Table 6: Area under the curve (AUC) for the detection of protein isoforms, for every method in each real dataset, computed on the subset of protein isoforms from multi-isoform genes (i.e., genes with more than one expressed isoform).

1.5 Real data - Robustness to input data

<table>
<tr><th rowspan="2">Cell line</th><th rowspan="2">Metric</th><th colspan="3">IsoBayes</th><th colspan="3">IsoBayes_mRNA</th></tr>
<tr><th>OpenMS</th><th>MM PSM</th><th>MM int</th><th>OpenMS</th><th>MM PSM</th><th>MM int</th></tr>
<tr><td rowspan="2">jurkat</td><td>AUC</td><td>0.79</td><td>0.81</td><td>0.81</td><td>0.84</td><td>0.86</td><td>0.86</td></tr>
<tr><td>Correlation</td><td>0.57</td><td>0.58</td><td>0.6</td><td>0.64</td><td>0.64</td><td>0.66</td></tr>
<tr><td rowspan="2">WTC-11</td><td>AUC</td><td>0.78</td><td>0.81</td><td>0.81</td><td>0.86</td><td>0.88</td><td>0.88</td></tr>
<tr><td>Correlation</td><td>0.49</td><td>0.49</td><td>0.44</td><td>0.64</td><td>0.64</td><td>0.58</td></tr>
</table>

Supplementary Table 7: Area under the curve (AUC) for the detection of protein isoforms, and correlation between log10 estimated protein isoform abundances (i.e., log10(abundance + 1)), and those found in the validation set. Values represent averages across the proteases of the jurkat and WTC-11 datasets. Results refer to *IsoBayes* and *IsoBayes_mRNA*, computed on three input data: i) PSM counts from *OpenMS*' *Percolator* ("OpenMS" column); ii) PSM counts from *MetaMorpheus* ("MM PSM" column); iii) peptide intensities from *MetaMorpheus*("MM int" column). Note that numbers slightly differ with respect to other Tables; this is because, here, we focus on the isoforms that are in common across the three data types, i.e., with at least 1 detected (shared or unique) peptide. This ensures a fair comparison across inputs.

1.6 Real data - PEP *vs.* FDR mode

Input data	Cell line	Metric	*IsoBayes*		*IsoBayes_ mRNA*	
			PEP mode	*FDR mode*	*PEP mode*	*FDR mode*
MM PSM	jurkat	AUC	0.82	0.81	0.86	0.85
		Correlation	0.60	0.60	0.65	0.65
	WTC-11	AUC	0.81	0.81	0.88	0.87
		Correlation	0.51	0.51	0.63	0.64
MM int	jurkat	AUC	0.82	0.81	0.87	0.86
		Correlation	0.62	0.61	0.68	0.67
	WTC-11	AUC	0.81	0.81	0.88	0.87
		Correlation	0.48	0.45	0.61	0.59

Supplementary Table 8: Area under the curve (AUC) for the detection of protein isoforms, and correlation between log10 estimated protein isoform abundances (i.e., log10(abundance + 1)), and those found in the validation set. Values represent averages across the proteases of the jurkat and WTC-11 datasets. Results refer to *IsoBayes* and *IsoBayes_ mRNA*, based the PEP and FDR modes. Methods were fit on both PSM counts ("MM PSM" rows), and peptide intensities ("MM int" rows), computed via *MetaMorpheus*.

Input data	Cell line	Metric	*IsoBayes*		*IsoBayes_ mRNA*	
			PEP mode	*FDR mode*	*PEP mode*	*FDR mode*
MM PSM	jurkat	Runtime (mins)	0.9	0.4	0.9	0.4
		Memory (GB)	5.4	3.3	5.0	3.3
	WTC-11	Runtime (mins)	4.1	0.7	4.1	0.7
		Memory (GB)	5.7	3.5	6.5	5.2
MM int	jurkat	Runtime (mins)	0.9	0.4	0.9	0.4
		Memory (GB)	5.5	3.3	5.9	3.4
	WTC-11	Runtime (mins)	4.4	0.6	4.3	0.7
		Memory (GB)	6.4	3.9	6.5	4.9

Supplementary Table 9: Runtime (in minutes) and memory (in Gigabytes) for *IsoBayes* and *IsoBayes_ mRNA*, based the PEP and FDR modes. Values represent averages across the proteases of the jurkat and WTC-11 datasets. Methods were fit on both PSM counts ("MM PSM" rows), and peptide intensities ("MM int" rows), computed via *MetaMorpheus*.

2 Supplementary Figures

2.1 All protein isoforms

2.1.1 Isoform-level results

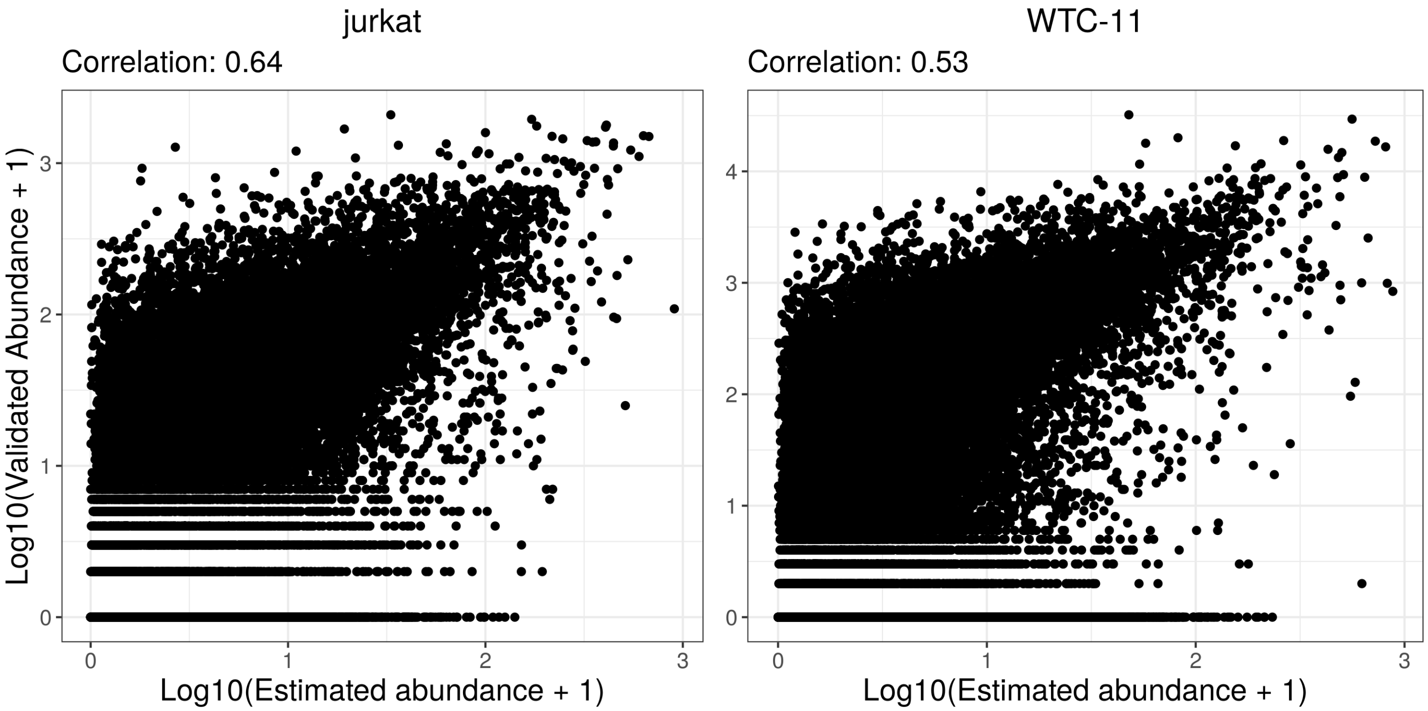

Supplementary Figure 1: Scatterplot for the log10 protein isoform abundances (i.e., log10(abundance + 1)), estimated from *IsoBayes* (x axis), and found in the validation set (y axis). In each cell line, we considered results from all protesease. Left: *jurkat* dataset; right: *WTC-11* dataset.

2.1.2 Gene-level results

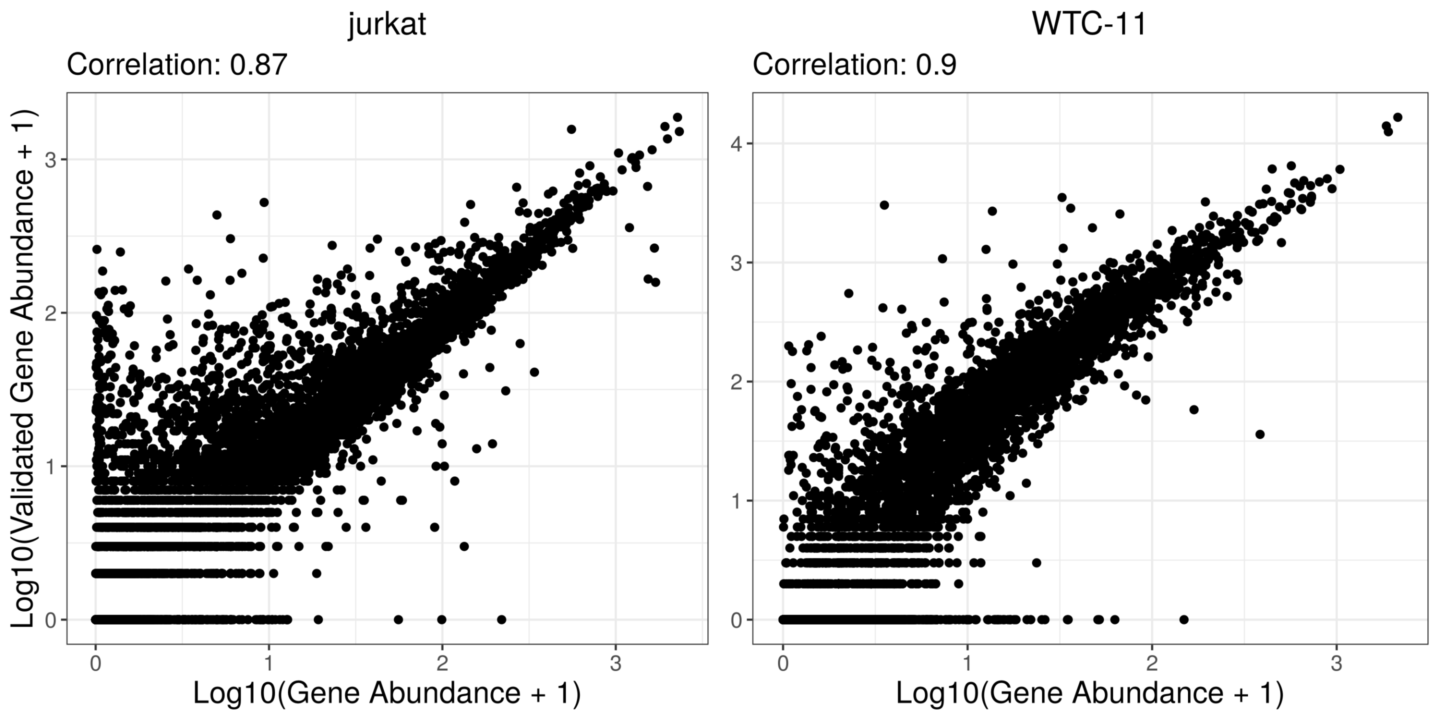

Supplementary Figure 2: Scatterplot for the log10 protein gene abundances (i.e., log10(abundance + 1)), estimated from *IsoBayes_mRNA* (x axis), and found in the validation set (y axis). In each cell line, we considered results from all protesease. Left: *jurkat* dataset; right: *WTC-11* dataset.

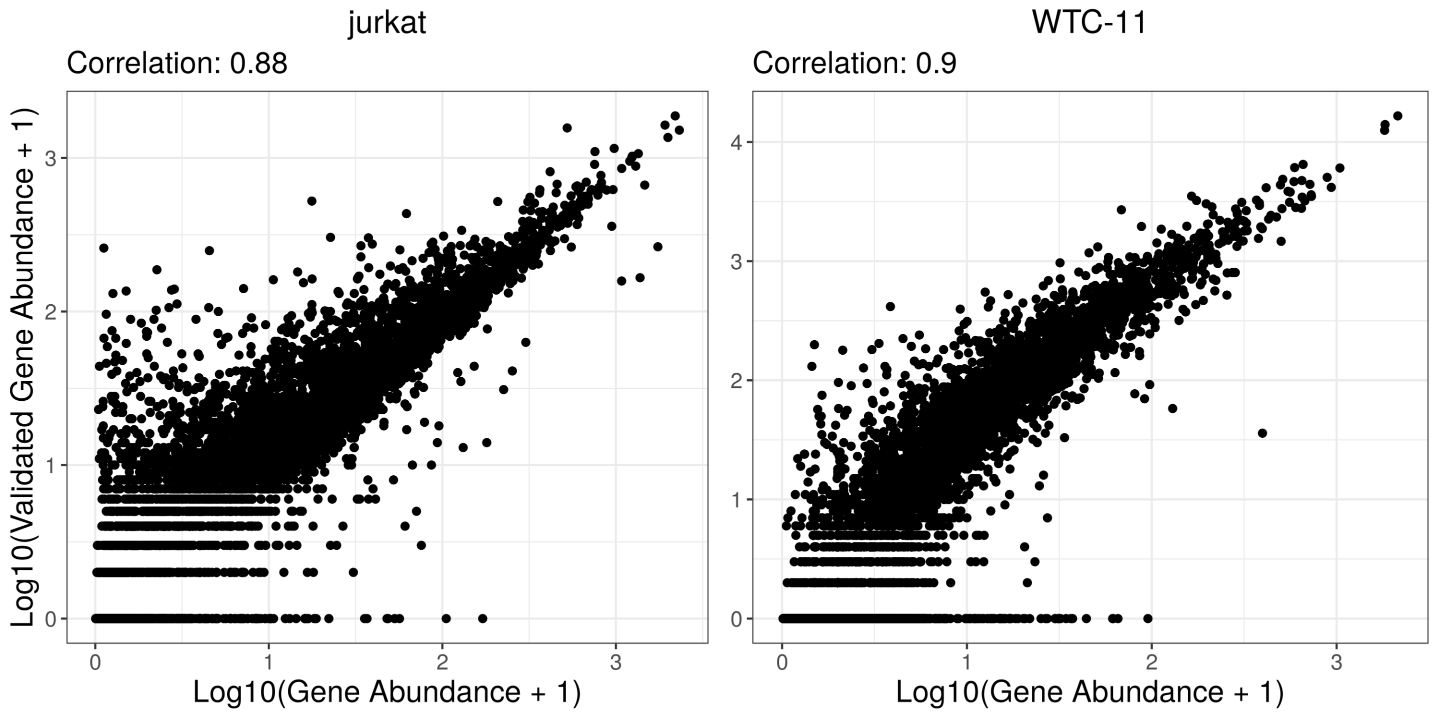

Supplementary Figure 3: Scatterplot for the log10 protein gene abundances (i.e., log10(abundance + 1)), estimated from *IsoBayes* (x axis), and found in the validation set (y axis). In each cell line, we considered results from all protesease. Left: *jurkat* dataset; right: *WTC-11* dataset.

2.1.3 log2-FCs

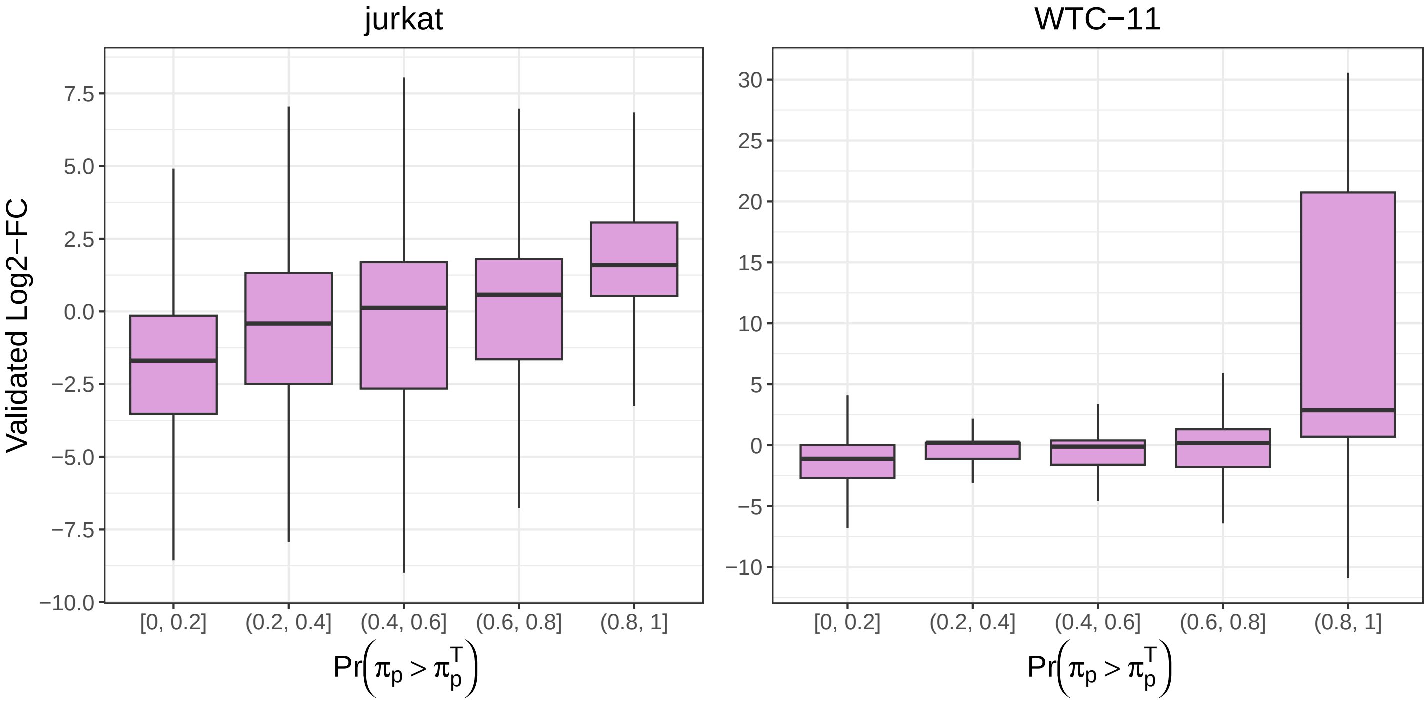

Supplementary Figure 4: Boxplot of the stabilized log2-FCs between protein and transcript relative abundances, identified in the validated set, stratified based on the probability, estimated by *IsoBayes_mRNA*, that isoform relative abundances are higher at the protein- than at then transcript-level. In each cell line, we considered results from all protesease. Left: *jurkat* dataset; right: *WTC-11* dataset.

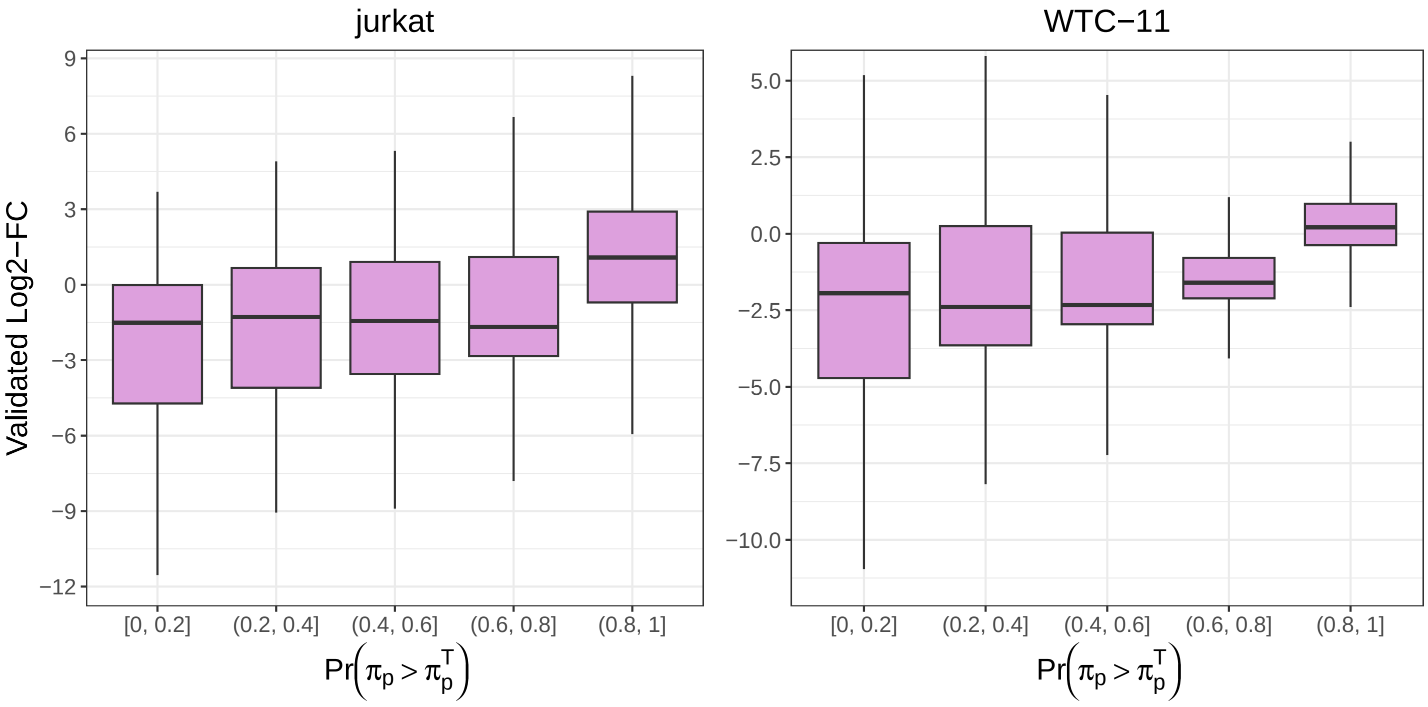

Supplementary Figure 5: Boxplot of the stabilized log2-FCs between protein and transcript relative abundances, identified in the validated set, stratified based on the probability, estimated by *IsoBayes*, that isoform relative abundances are higher at the protein- than at then transcript-level. In each cell line, we considered results from all protesease. Left: *jurkat* dataset; right: *WTC-11* dataset.

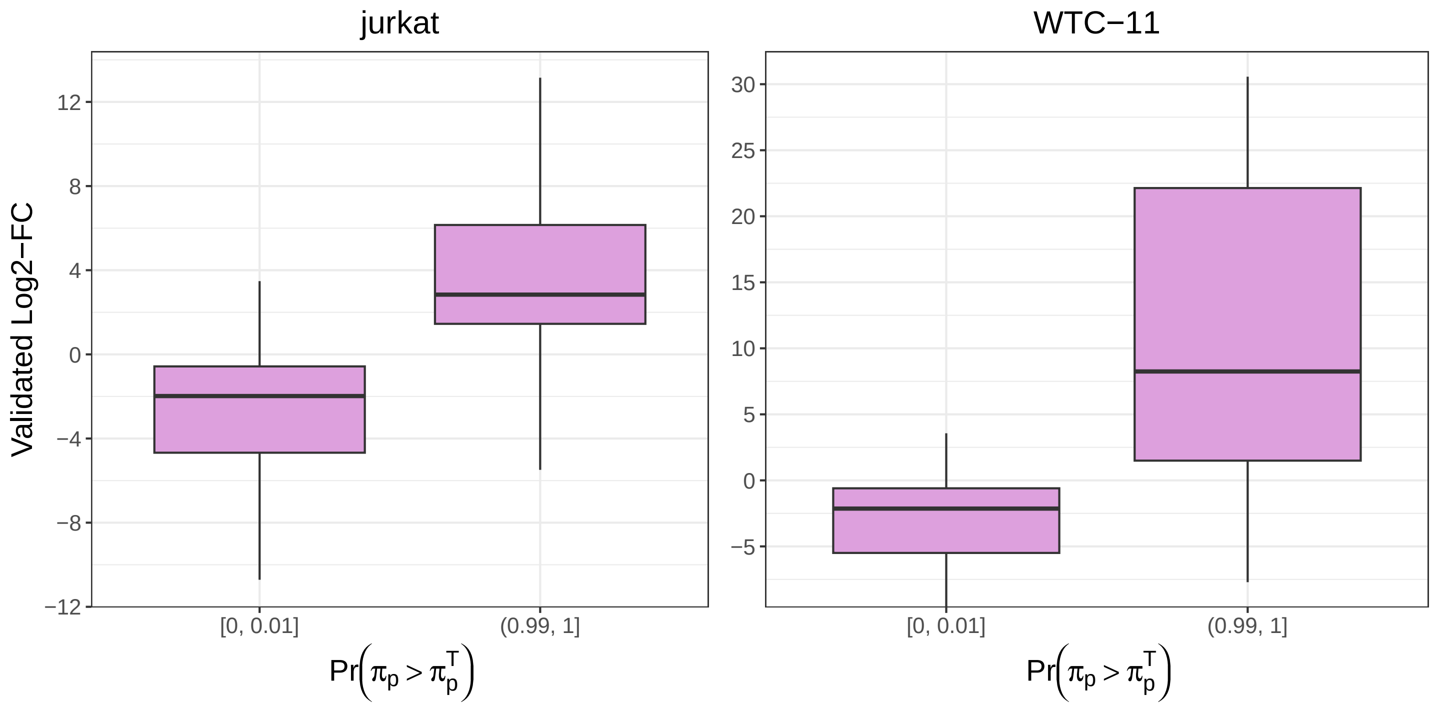

Supplementary Figure 6: Boxplot of the stabilized log2-FCs between protein and transcript relative abundances, identified in the validated set, stratified based on the probability, estimated by *IsoBayes*, that isoform relative abundances are higher at the protein- than at then transcript-level. Small estimated probabilities (below 0.01) are mainly associated to negative log2-FCs in the validation set; conversely, large estimated probabilities (above 0.99) typically lead to positive log2-FCs in the validation set. In each cell line, we considered results from all protesease. Left: *jurkat* dataset; right: *WTC-11* dataset.

2.1.4 Memory usage

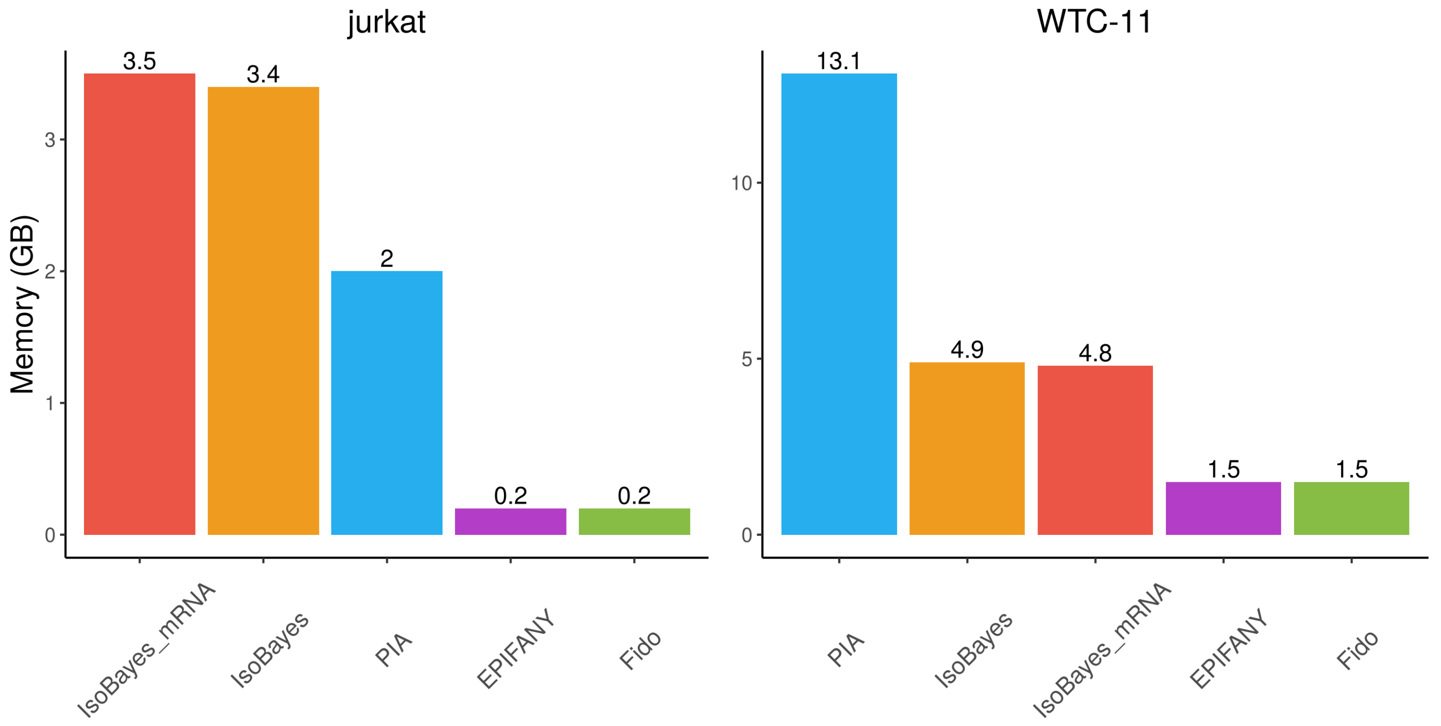

Supplementary Figure 7: Average (across proteases) of the maximum memory required by each method, expressed in gigabytes (GB). Left: *jurkat* dataset; right: *WTC-11* dataset.

2.2 Isoforms without unique peptides

2.2.1 Isoform-level results

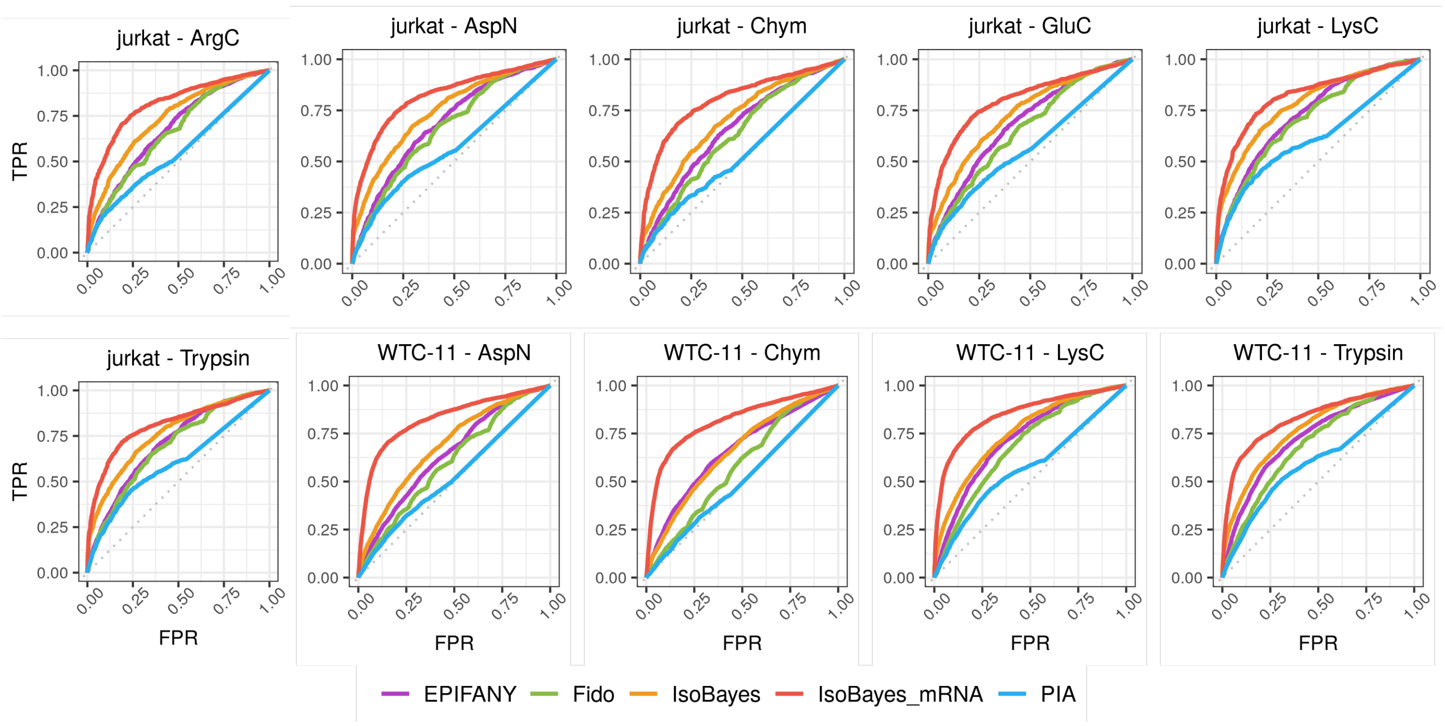

Supplementary Figure 8: Receiver operating characteristic (ROC) curves for the detection of protein isoforms in each real dataset, computed on the subset of protein isoforms solely associated to shared peptides.

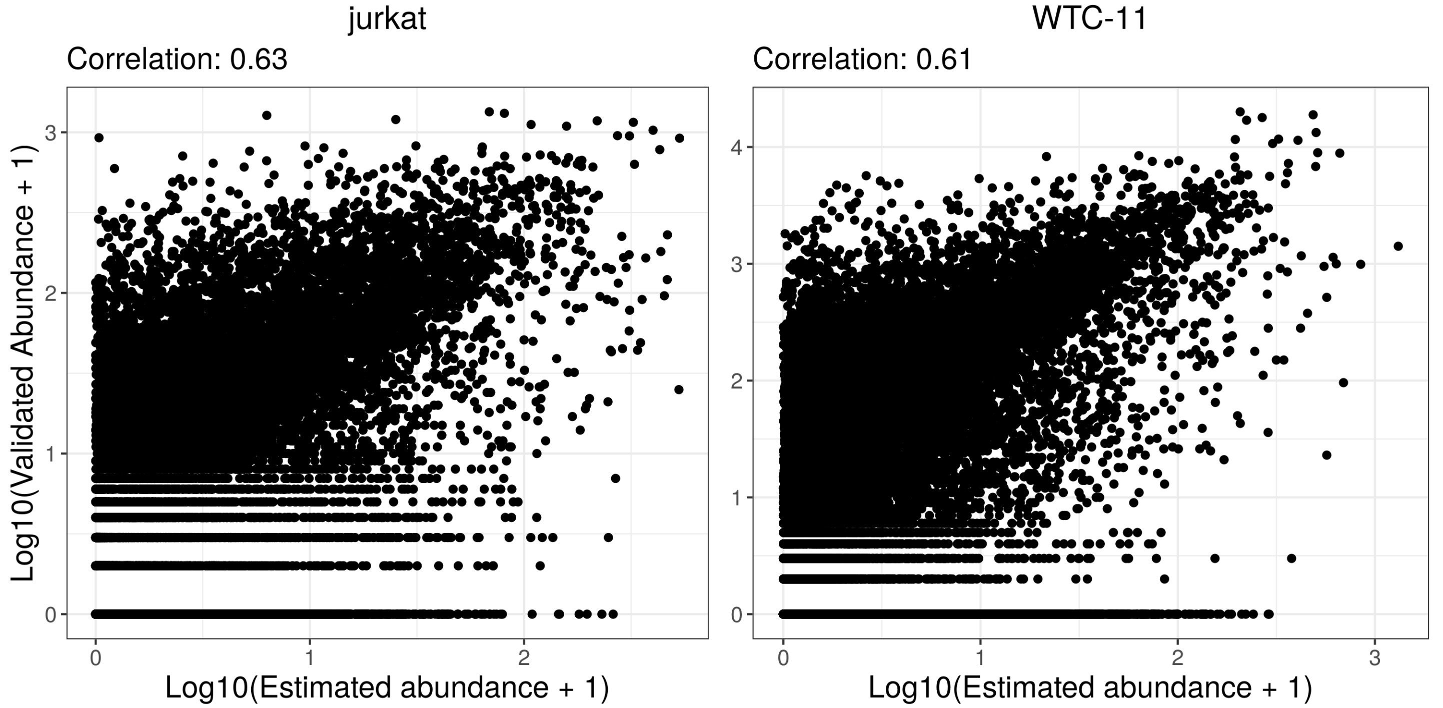

Supplementary Figure 9: Scatterplot for the log10 protein isoform abundances (i.e., log10(abundance + 1)), estimated from *IsoBayes_mRNA* (x axis), and found in the validation set (y axis), computed on the subset of protein isoforms solely associated to shared peptides. In each cell line, we considered results from all protesease. Left: *jurkat* dataset; right: *WTC-11* dataset.

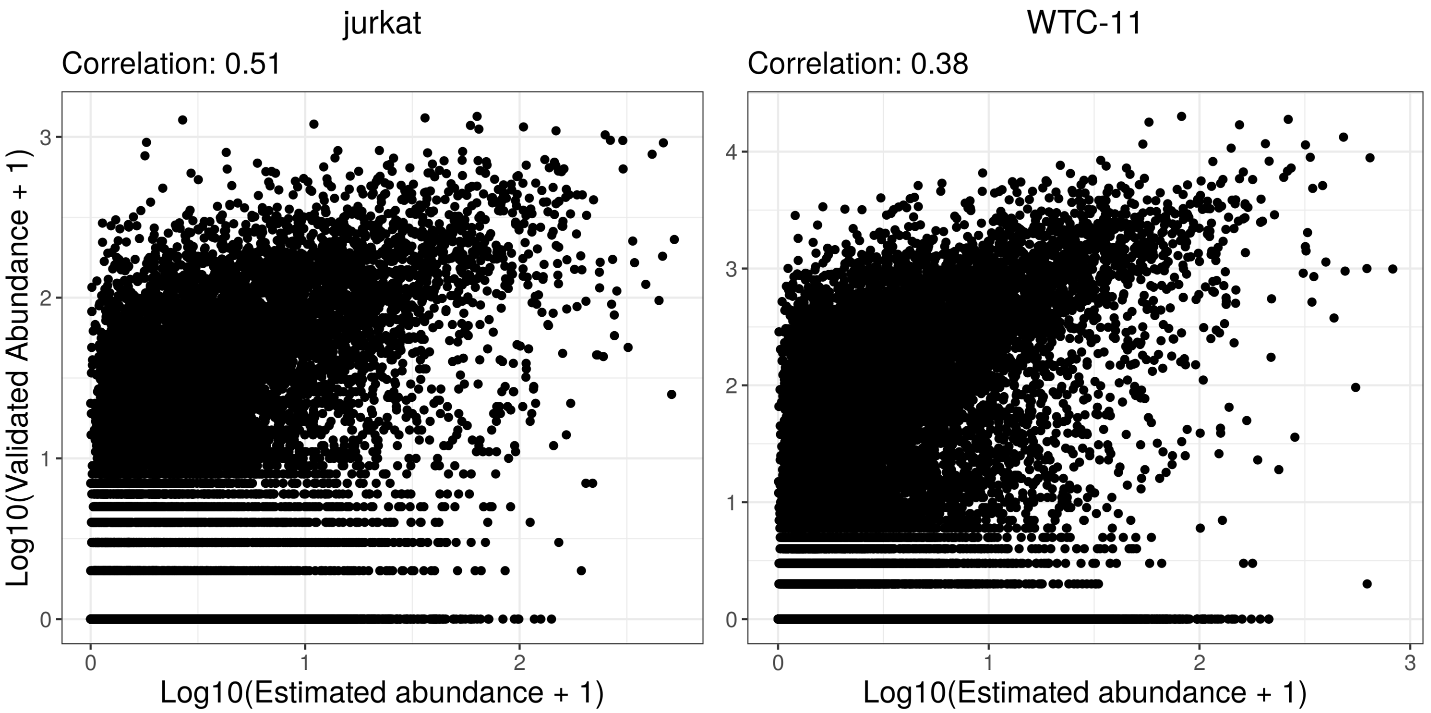

Supplementary Figure 10: Scatterplot for the log10 protein isoform abundances (i.e., log10(abundance + 1)), estimated from *IsoBayes* (x axis), and found in the validation set (y axis), computed on the subset of protein isoforms solely associated to shared peptides. In each cell line, we considered results from all protesease. Left: *jurkat* dataset; right: *WTC-11* dataset.

2.2.2 log2-FCs

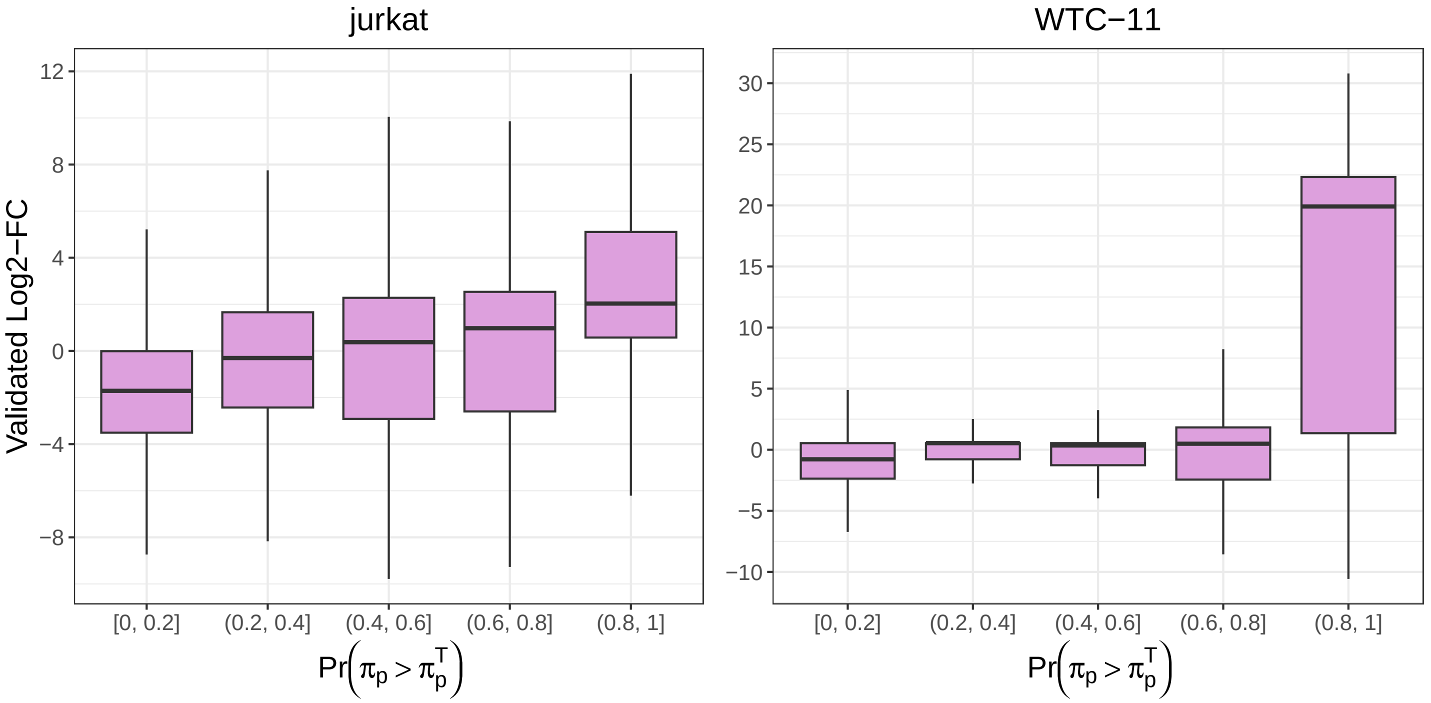

Supplementary Figure 11: Boxplot of the stabilized log2-FCs between protein and transcript relative abundances, identified in the validated set, stratified based on the probability, estimated by *IsoBayes_mRNA*, that isoform relative abundances are higher at the protein- than at then transcript-level, computed on the subset of protein isoforms solely associated to shared peptides. In each cell line, we considered results from all protesease. Left: *jurkat* dataset; right: *WTC-11* dataset.

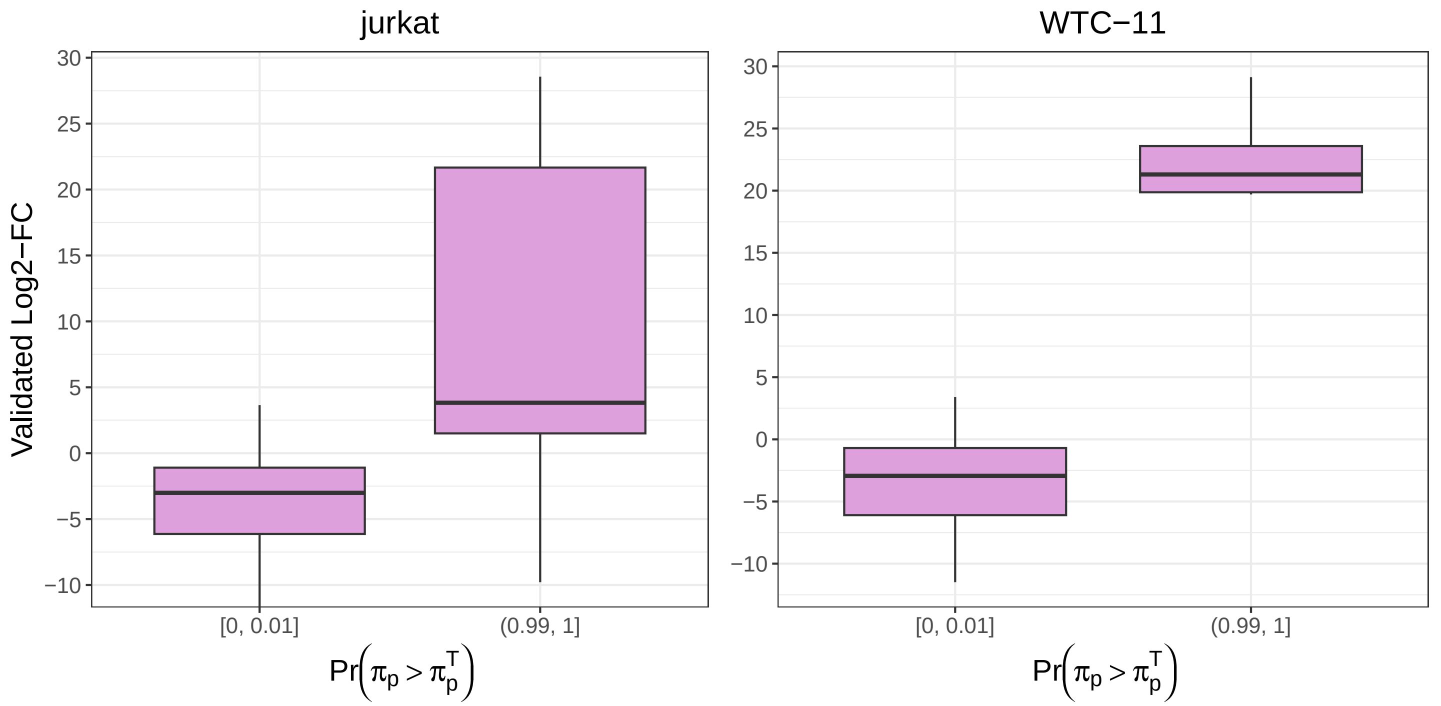

Supplementary Figure 12: Boxplot of the stabilized log2-FCs between protein and transcript relative abundances, identified in the validated set, stratified based on the probability, estimated by *IsoBayes_mRNA*, that isoform relative abundances are higher at the protein- than at then transcript-level, computed on the subset of protein isoforms solely associated to shared peptides. Small estimated probabilities (below 0.01) are mainly associated to negative log2-FCs in the validation set; conversely, large estimated probabilities (above 0.99) typically lead to positive log2-FCs in the validation set. In each cell line, we considered results from all protesease. Left: *jurkat* dataset; right: *WTC-11* dataset.

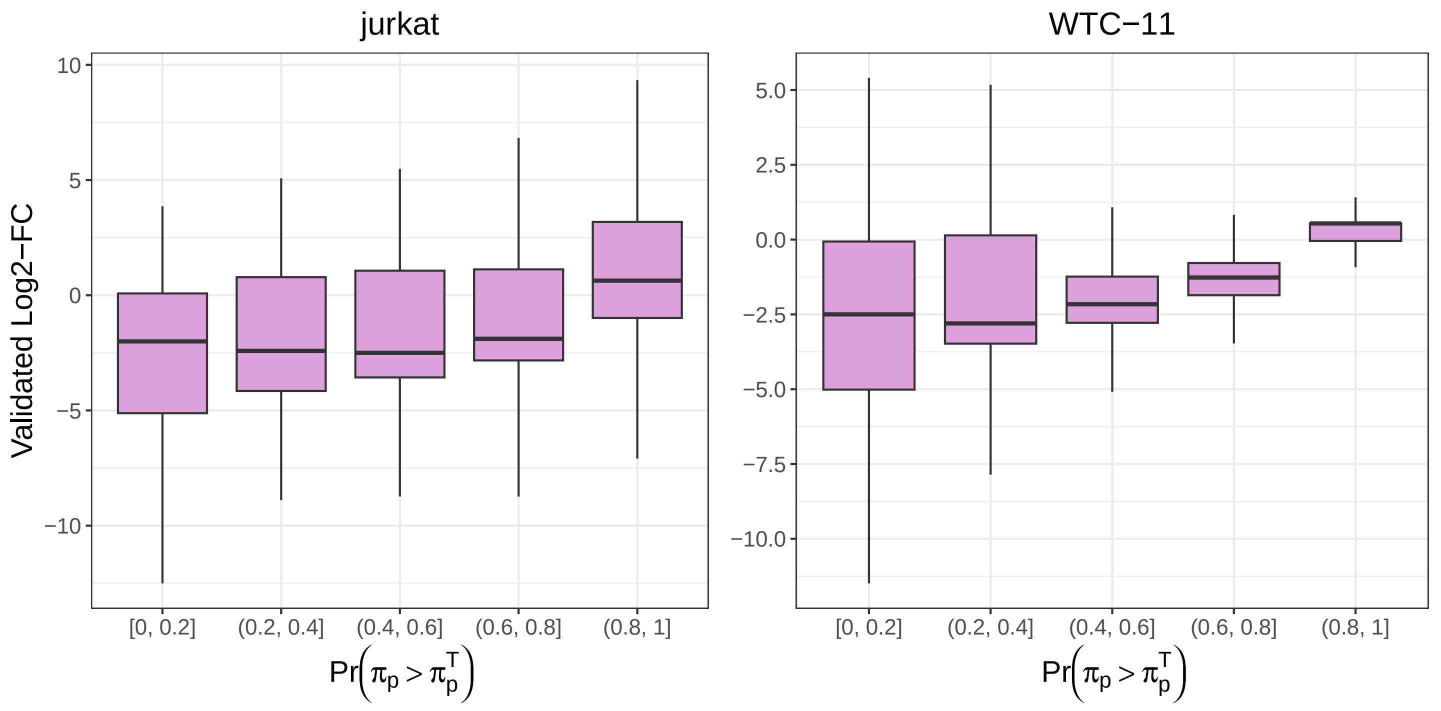

Supplementary Figure 13: Boxplot of the stabilized log2-FCs between protein and transcript relative abundances, identified in the validated set, stratified based on the probability, estimated by *IsoBayes*, that isoform relative abundances are higher at the protein- than at then transcript-level, computed on the subset of protein isoforms solely associated to shared peptides. In each cell line, we considered results from all protesease. Left: *jurkat* dataset; right: *WTC-11* dataset.

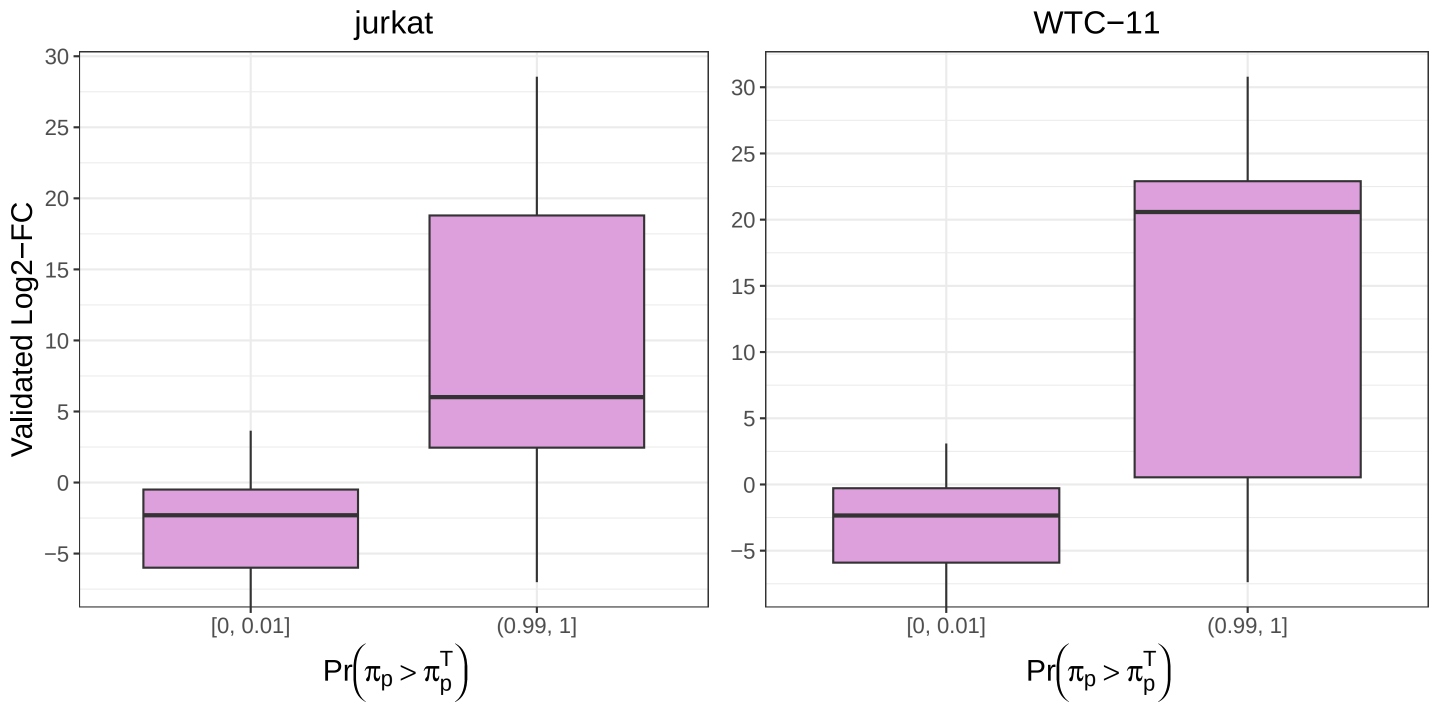

Supplementary Figure 14: Boxplot of the stabilized log2-FCs between protein and transcript relative abundances, identified in the validated set, stratified based on the probability, estimated by *IsoBayes*, that isoform relative abundances are higher at the protein- than at then transcript-level, computed on the subset of protein isoforms solely associated to shared peptides. Small estimated probabilities (below 0.01) are mainly associated to negative log2-FCs in the validation set; conversely, large estimated probabilities (above 0.99) typically lead to positive log2-FCs in the validation set. In each cell line, we considered results from all protesease. Left: *jurkat* dataset; right: *WTC-11* dataset.

2.3 Robustness to input data

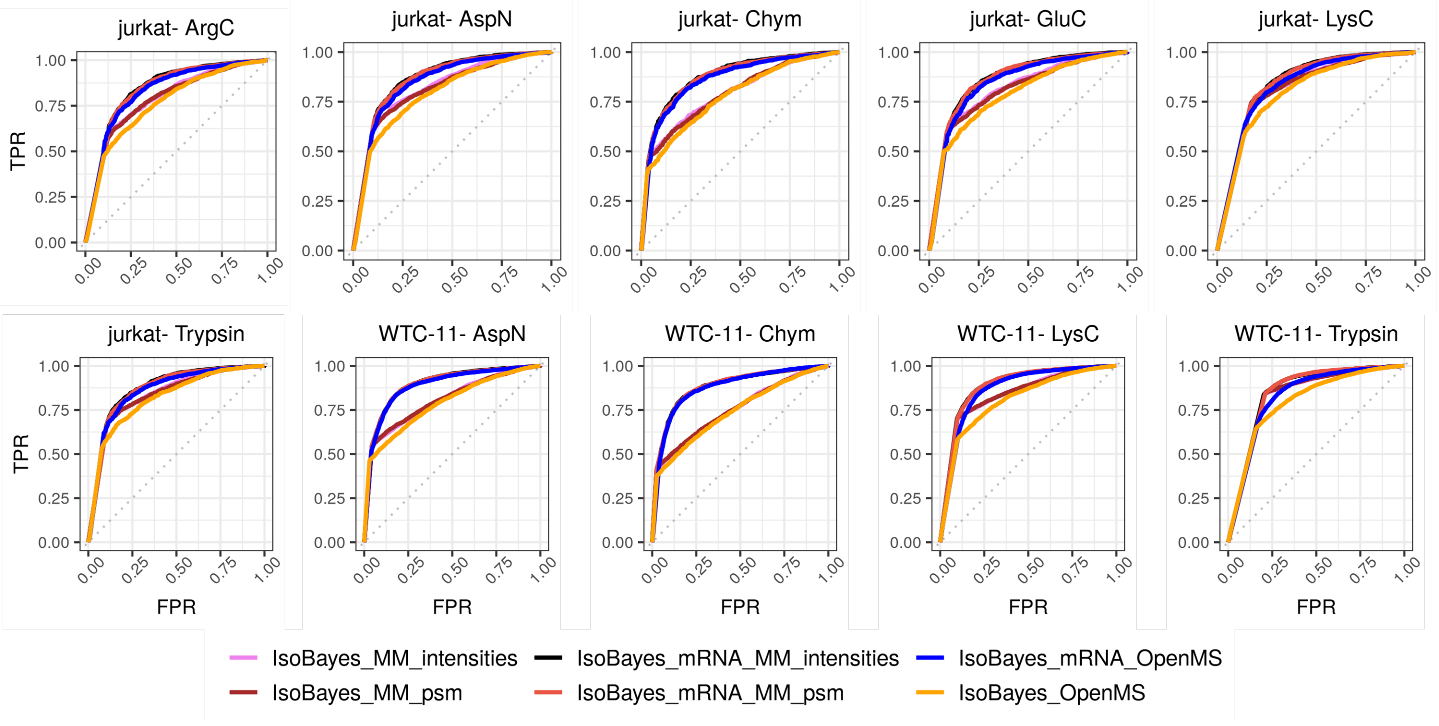

Supplementary Figure 15: Receiver operating characteristic (ROC) curves for the detection of protein isoforms in each real dataset, for *IsoBayes* and *IsoBayes_mRNA*, computed on three input data: i) PSM counts from *OpenMS' Percolator*; ii) PSM counts from *MetaMorpheus*; iii) peptide intensities from *MetaMorpheus*. Since slightly different peptides are detected between *MetaMorpheus* and *Percolator*, for a fair comparison, here we removed the minority of isoforms which can be analyzed with one tool only, and not with the other one.

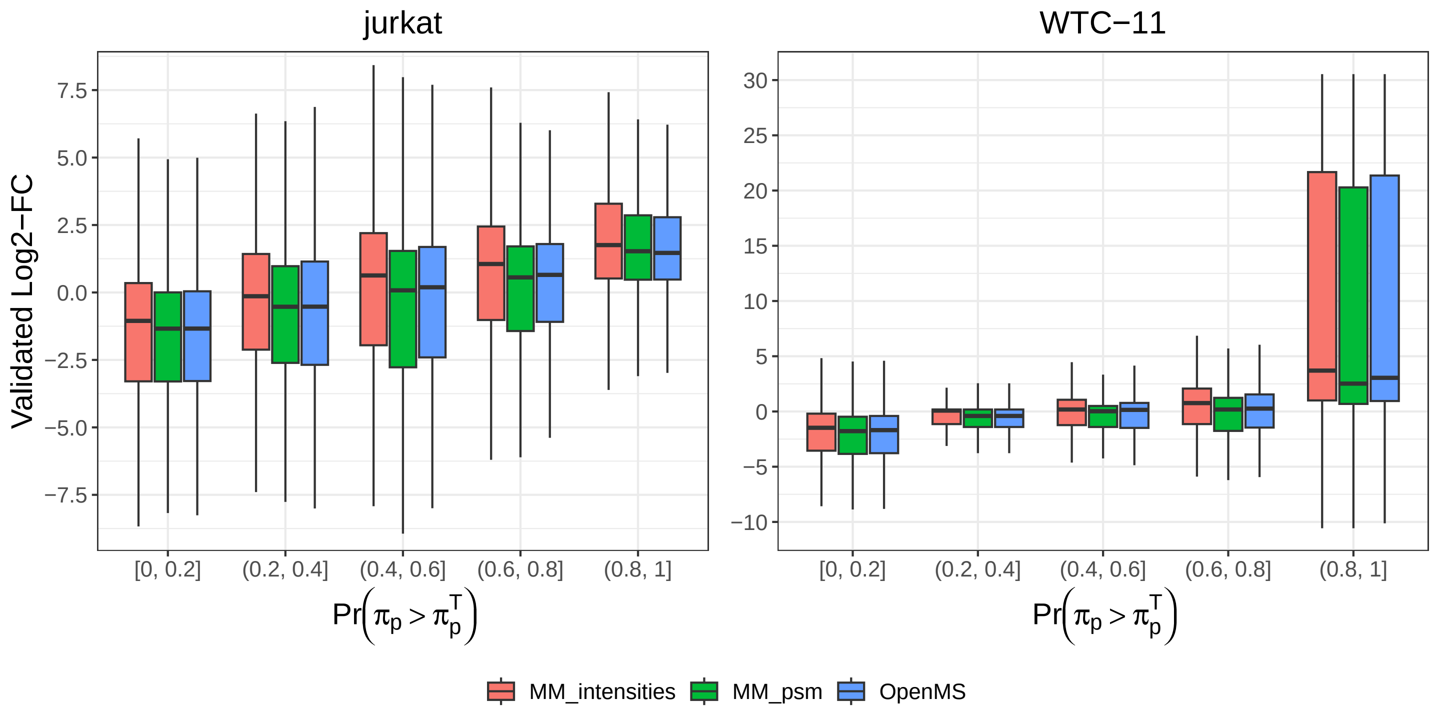

Supplementary Figure 16: Boxplot of the stabilized log2-FCs between protein and transcript relative abundances, identified in the validated set, stratified based on the probability, estimated by *IsoBayes_mRNA*, that isoform relative abundances are higher at the protein- than at then transcript-level. The three colours refer to the three inputs: i) PSM counts from *OpenMS*' *Percolator* ("OpenMS"); ii) PSM counts from *MetaMorpheus* ("MM_PSM"); iii) peptide intensities from *MetaMorpheus*("MM_intensities"). In each cell line, we considered results from all protesease. Left: *jurkat* dataset; right: *WTC-11* dataset. Since slightly different peptides are detected between *MetaMorpheus* and *Percolator*, for a fair comparison, here we removed the minority of isoforms which can be analyzed with one tool only, and not with the other one.

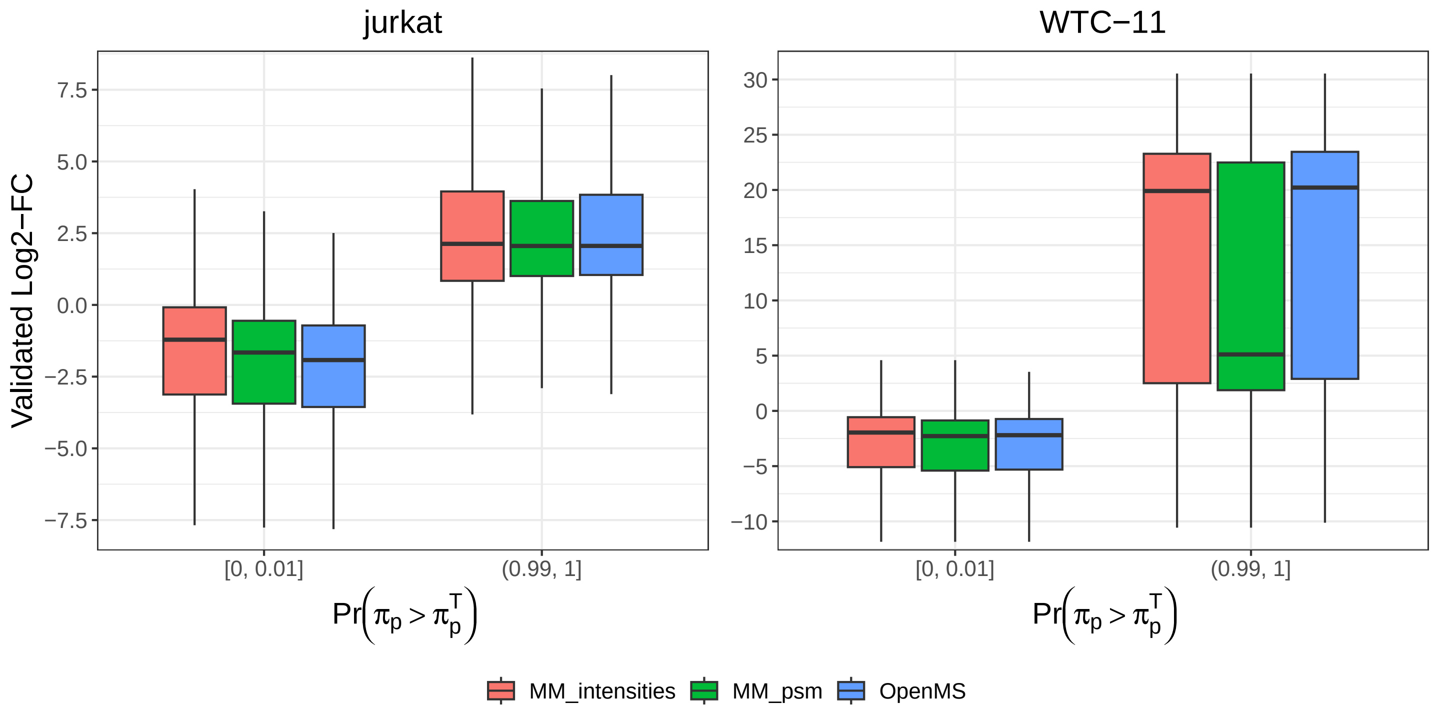

Supplementary Figure 17: Boxplot of the stabilized log2-FCs between protein and transcript relative abundances, identified in the validated set, stratified based on the probability, estimated by *IsoBayes_mRNA*, that isoform relative abundances are higher at the protein- than at then transcript-level. The three colours refer to the three inputs: i) PSM counts from *OpenMS*' *Percolator* ("OpenMS"); ii) PSM counts from *MetaMorpheus* ("MM_PSM"); iii) peptide intensities from *MetaMorpheus*("MM_intensities"). In each cell line, we considered results from all protesease. Left: *jurkat* dataset; right: *WTC-11* dataset. Since slightly different peptides are detected between *MetaMorpheus* and *Percolator*, for a fair comparison, here we removed the minority of isoforms which can be analyzed with one tool only, and not with the other one.

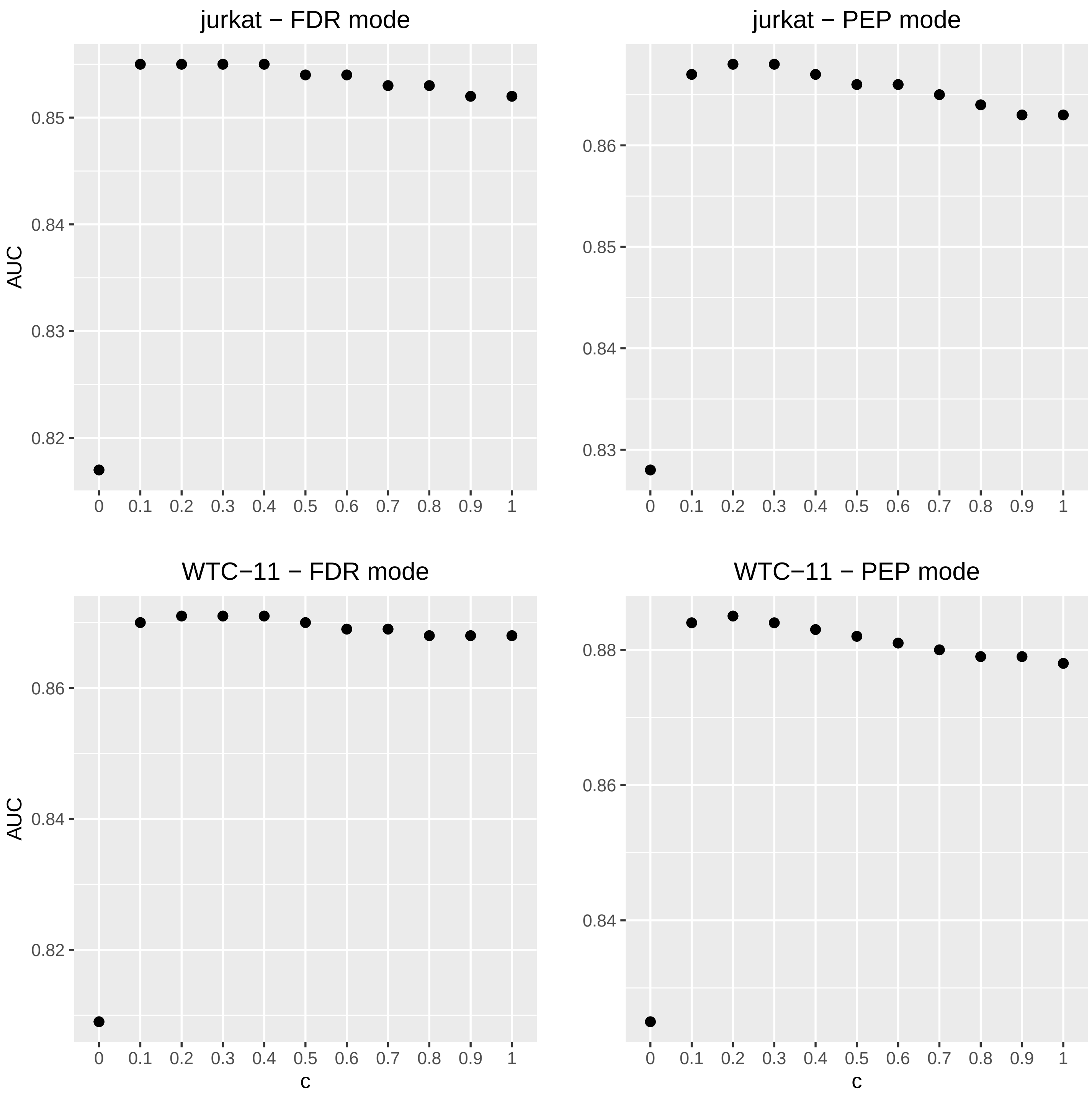

Supplementary Figure 18: Area under the curve (AUC) depending on the strength of the informative prior (mRNA relative abundances). On the x-axis, 0 indicates that mRNA was not used to formulate the informative prior (i.e., $\delta_1 = \cdots = \delta_P = 1$), while 1 denotes that mRNA and protein data have the same weight in the posterior distribution of $\pi|X, \delta$. By default, in *IsoBayes* "c" is set to 0.1, which in general provides the highest AUC in our benchmarks, as visible in the image. Top row: *jurkat* data; bottom row: *WTC-11* data. Left panels: PEP mode; right panels: FDR mode. AUC values represent averages across the AUC values obtained on the six *jurkat* and four *WTC-11* proteases.
